# 5-(4-Chloro­phen­yl)-1-(2,4-dichloro­phen­yl)-4-methyl-1*H*-pyrazole-3-carboxylic acid

**DOI:** 10.1107/S1600536808038105

**Published:** 2008-11-22

**Authors:** Wei Wang, Zheng Fang

**Affiliations:** a College of Life Sciences and Pharmaceutical Engineering, Nanjing University of Technolgy, Xinmofan Road No. 5 Nanjing, Nanjing 210009, People’s Republic of China

## Abstract

The asymmetric unit of the title compound, C_17_H_11_Cl_3_N_2_O_2_, contains two independent mol­ecules; the pyrazole rings are oriented with respect to the chloro­phenyl and dichloro­phenyl rings at dihedral angles of 43.00 (3) and 65.06 (4)°, respectively, in one mol­ecule, and 51.17 (3) and 69.99 (3)°, respectively, in the other. Pairs of inter­molecular O—H⋯O hydrogen bonds link the mol­ecules into dimers. In the crystal structure, there are π–π contacts between the pyrazole rings and dichloro­phenyl rings [centroid–centroid distances = 3.859 (3) and 3.835 (3) Å].

## Related literature

For bond-length data, see: Allen *et al.* (1987 ▶). For the chemical background, see: Tang *et al.* (2007 ▶).
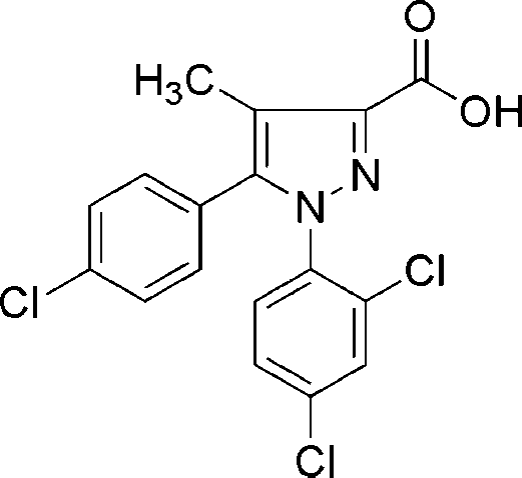


## Experimental

### 

#### Crystal data

C_17_H_11_Cl_3_N_2_O_2_
*M* *_r_* = 381.63
Monoclinic, 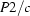

*a* = 13.192 (3) Å
*b* = 8.8170 (18) Å
*c* = 30.012 (6) Å
β = 102.42 (3)°
*V* = 3409.1 (13) Å^3^
*Z* = 8
Mo *K*α radiation
μ = 0.55 mm^−1^
*T* = 294 (2) K
0.30 × 0.20 × 0.10 mm

#### Data collection

Enraf–Nonius CAD-4 diffractometer
Absorption correction: ψ scan (North *et al.*, 1968 ▶) *T*_min_ = 0.853, *T*_max_ = 0.947
6479 measured reflections
6190 independent reflections
2893 reflections with *I* > 2σ(*I*)
*R*_int_ = 0.038
3 standard reflections frequency: 120 min intensity decay: 1%

#### Refinement

*R*[*F* ^2^ > 2σ(*F* ^2^)] = 0.072
*wR*(*F* ^2^) = 0.181
*S* = 0.99
6190 reflections
433 parameters
H-atom parameters constrained
Δρ_max_ = 0.33 e Å^−3^
Δρ_min_ = −0.28 e Å^−3^

### 

Data collection: *CAD-4 Software* (Enraf–Nonius, 1989 ▶); cell refinement: *CAD-4 Software*; data reduction: *XCAD4* (Harms & Wocadlo, 1995 ▶); program(s) used to solve structure: *SHELXS97* (Sheldrick, 2008 ▶); program(s) used to refine structure: *SHELXL97* (Sheldrick, 2008 ▶); molecular graphics: *PLATON* (Spek, 2003 ▶); software used to prepare material for publication: *SHELXL97*.

## Supplementary Material

Crystal structure: contains datablocks global, I. DOI: 10.1107/S1600536808038105/hk2574sup1.cif

Structure factors: contains datablocks I. DOI: 10.1107/S1600536808038105/hk2574Isup2.hkl

Additional supplementary materials:  crystallographic information; 3D view; checkCIF report

## Figures and Tables

**Table 1:** Hydrogen-bond geometry (Å, °)

*D*—H⋯*A*	*D*—H	H⋯*A*	*D*⋯*A*	*D*—H⋯*A*
O2—H2*A*⋯O4	0.85	1.74	2.564 (7)	163
O3—H3*B*⋯O1	0.85	1.89	2.723 (6)	165

Symmetry codes: (i) 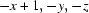; (ii) 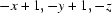.

**Fig. 1.**
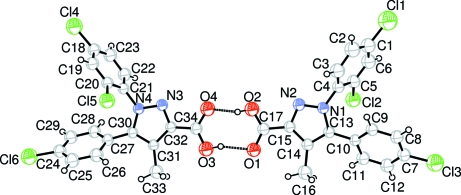
The molecular structure of the title molecule, with the atom-numbering scheme.

## supplementary crystallographic information

### Comment

Some derivatives of benzoic acid are important chemical materials. We report herein the crystal structure of the title compound.

The asymmetric unit of the title compound contains two crystallographically independent molecules (Fig. 1). The bond lengths (Allen *et al.*, 1987) and angles are within normal ranges. Rings A (C1-C6), B (C7-C12), C (N1/N2/C13-C15) and D (C18-C23), E (C24-C29), F (N3/N4/C30-C32) are, of course, planar and they are oriented at dihedral angles of A/B = 58.42 (3)°, A/C = 65.06 (4), B/C = 43.00 (3)° and D/E = 57.07 (4)°, D/F = 69.99 (3)°, E/F = 51.17 (3)°. The intramolecuar O-H···O hydrogen bonds (Table 1) link the molecules (Fig. 1), in which they may be effective in the stabilization of the structure.

In the crystal structure, there are π-π contacts between the pyrazole rings and dichlorophenyl rings, Cg1—Cg2^i^ and Cg3—Cg5^ii^ [symmetry codes: (i) x + 1, y, z; (ii) 1 - x, y, 1/2 -z, where Cg1, Cg2, Cg3 and Cg5 are centroids of the rings C (N1/N2/C13-C15), F (N3/N4/C30-C32), A (C3-C8) and D (C18-C23), respectively] may stabilize the structure, with centroid-centroid distances of 3.859 (3) Å and 3.835 (3) Å, respectively. There also exist C—H···π contacts (Table 1) between the methyl groups and the chlorophenyl rings.

### Experimental

For the preparation of the title compound, 2,4-dichlorophenylhydrazine hydrochloride (13.3 g) diluted in ethanol (20 ml) is added to ethyl 4-(4-chlorophenyl)-3-methyl-2,4-dioxobutanoate (17.6 g) diluted in toluene (50 ml) and the mixture is stirred for 18 h at room temperature. Without isolating the hydrazone, paratoluenesulfonic acid (0.56 g) is added, and the ternary azeotrope (water, ethanol, toluene) is distilled. Toluene reflux is continued for 1 h and the reaction mixture is cooled to room temperature. The insoluble material is filtered off. The solvents are removed under reduced pressure to give an oil. KOH (8.1 g) in pellets are added to a solution of the oil obtained in the previous step in MeOH (100 ml). The mixture is left for 1 h at room temperature and the solvents are decanted into water (200 ml) at 333 K. Hydrochloric acid is then added to the aqueous phase until pH = 1.5. The colorless crystals formed are filtered off, washed with water and dried under vaccum to give the expected product (yield; 9.9 g). Crystals suitable for X-ray analysis were obtained by slow evaporation of an acetic acid solution.

### Refinement

H atoms were positioned geometrically, with O-H = 0.85 Å (for OH) and C-H = 0.93 and 0.96 Å for aromatic and methyl H, respectively, and constrained to ride on their parent atoms with U_iso_(H) = xU_eq_(C,O), where x = 1.2 for aromatic H and x = 1.5 for all other H atoms.

### Crystal data

**Table:**

C_17_H_11_Cl_3_N_2_O_2_	*F*_000_ = 1552
*M**_r_* = 381.63	*D*_x_ = 1.487 Mg m^−^^3^
Monoclinic, *P*2/*c*	Mo *K*α radiation λ = 0.71073 Å
Hall symbol: -P 2yc	Cell parameters from 25 reflections
*a* = 13.192 (3) Å	θ = 10–13º
*b* = 8.8170 (18) Å	µ = 0.55 mm^−^^1^
*c* = 30.012 (6) Å	*T* = 294 (2) K
β = 102.42 (3)º	Block, colorless
*V* = 3409.1 (13) Å^3^	0.30 × 0.20 × 0.10 mm
*Z* = 8	

### Data collection

**Table:**

Enraf–Nonius CAD-4 diffractometer	*R*_int_ = 0.038
Radiation source: fine-focus sealed tube	θ_max_ = 25.3º
Monochromator: graphite	θ_min_ = 1.4º
*T* = 294(2) K	*h* = 0→15
ω/2θ scans	*k* = 0→10
Absorption correction: ψ scan(North *et al.*, 1968)	*l* = −36→36
*T*_min_ = 0.853, *T*_max_ = 0.947	3 standard reflections
6479 measured reflections	every 120 min
6190 independent reflections	intensity decay: 1%
2893 reflections with *I* > 2σ(*I*)	

### Refinement

**Table:**

Refinement on *F*^2^	Secondary atom site location: difference Fourier map
Least-squares matrix: full	Hydrogen site location: inferred from neighbouring sites
*R*[*F*^2^ > 2σ(*F*^2^)] = 0.072	H-atom parameters constrained
*wR*(*F*^2^) = 0.181	*w* = 1/[σ^2^(*F*_o_^2^) + (0.06*P*)^2^ + 5*P*] where *P* = (*F*_o_^2^ + 2*F*_c_^2^)/3
*S* = 0.99	(Δ/σ)_max_ < 0.001
6190 reflections	Δρ_max_ = 0.33 e Å^−^^3^
433 parameters	Δρ_min_ = −0.28 e Å^−^^3^
Primary atom site location: structure-invariant direct methods	Extinction correction: none

### Special details

**Table:**

Geometry. All e.s.d.'s (except the e.s.d. in the dihedral angle between two l.s. planes) are estimated using the full covariance matrix. The cell e.s.d.'s are taken into account individually in the estimation of e.s.d.'s in distances, angles and torsion angles; correlations between e.s.d.'s in cell parameters are only used when they are defined by crystal symmetry. An approximate (isotropic) treatment of cell e.s.d.'s is used for estimating e.s.d.'s involving l.s. planes.
Refinement. Refinement of *F*^2^ against ALL reflections. The weighted *R*-factor *wR* and goodness of fit *S* are based on *F*^2^, conventional *R*-factors *R* are based on *F*, with *F* set to zero for negative *F*^2^. The threshold expression of *F*^2^ > σ(*F*^2^) is used only for calculating *R*-factors(gt) *etc*. and is not relevant to the choice of reflections for refinement. *R*-factors based on *F*^2^ are statistically about twice as large as those based on *F*, and *R*- factors based on ALL data will be even larger.

### Fractional atomic coordinates and isotropic or equivalent isotropic displacement parameters (Å^2^)

**Table:**

	*x*	*y*	*z*	*U*_iso_*/*U*_eq_	
Cl1	0.3235 (2)	0.6137 (2)	0.22847 (7)	0.1112 (9)	
Cl2	0.41462 (12)	0.2754 (2)	0.37992 (6)	0.0718 (5)	
Cl3	0.42824 (16)	0.8503 (2)	0.49709 (6)	0.0871 (7)	
Cl4	−0.31722 (17)	−1.1411 (2)	0.16706 (6)	0.0858 (6)	
Cl5	−0.38786 (12)	−0.7789 (2)	0.29976 (6)	0.0696 (5)	
Cl6	−0.45426 (14)	−1.3607 (2)	0.39707 (6)	0.0814 (6)	
O1	0.0459 (3)	−0.1244 (5)	0.43492 (13)	0.0571 (11)	
O2	0.0428 (3)	−0.1317 (5)	0.35970 (14)	0.0613 (12)	
H2A	0.0228	−0.2219	0.3629	0.092*	
O3	−0.0346 (3)	−0.4099 (5)	0.42547 (14)	0.0648 (13)	
H3B	−0.0097	−0.3220	0.4233	0.097*	
O4	−0.0241 (3)	−0.4055 (5)	0.35213 (14)	0.0636 (12)	
N1	0.1830 (3)	0.2661 (5)	0.37220 (15)	0.0398 (11)	
N2	0.1338 (3)	0.1348 (5)	0.36084 (15)	0.0442 (12)	
N3	−0.1099 (3)	−0.6749 (5)	0.33613 (15)	0.0414 (11)	
N4	−0.1607 (3)	−0.8069 (5)	0.33785 (14)	0.0387 (11)	
C1	0.2820 (7)	0.5083 (7)	0.2692 (2)	0.065 (2)	
C2	0.1789 (7)	0.4874 (8)	0.2669 (2)	0.071 (2)	
H2B	0.1303	0.5256	0.2423	0.085*	
C3	0.1464 (5)	0.4081 (7)	0.3017 (2)	0.0626 (18)	
H3A	0.0759	0.3967	0.3009	0.075*	
C4	0.2182 (4)	0.3469 (6)	0.33725 (18)	0.0435 (14)	
C5	0.3238 (5)	0.3634 (7)	0.3377 (2)	0.0519 (16)	
C6	0.3555 (6)	0.4459 (7)	0.3041 (2)	0.0642 (19)	
H6A	0.4258	0.4592	0.3049	0.077*	
C7	0.3576 (5)	0.6908 (7)	0.4752 (2)	0.0504 (16)	
C8	0.2851 (5)	0.7026 (7)	0.4350 (2)	0.0496 (16)	
H8A	0.2715	0.7959	0.4205	0.060*	
C9	0.2321 (4)	0.5733 (6)	0.4163 (2)	0.0446 (14)	
H9A	0.1836	0.5801	0.3888	0.053*	
C10	0.2507 (4)	0.4355 (6)	0.43793 (19)	0.0393 (13)	
C11	0.3227 (4)	0.4274 (7)	0.4789 (2)	0.0525 (16)	
H11A	0.3356	0.3349	0.4940	0.063*	
C12	0.3753 (5)	0.5553 (8)	0.4976 (2)	0.0563 (17)	
H12A	0.4226	0.5494	0.5254	0.068*	
C13	0.1975 (4)	0.2945 (6)	0.41780 (18)	0.0399 (13)	
C14	0.1523 (4)	0.1770 (6)	0.43595 (19)	0.0427 (14)	
C15	0.1141 (4)	0.0811 (6)	0.39951 (18)	0.0412 (14)	
C16	0.1457 (5)	0.1614 (7)	0.4862 (2)	0.0612 (18)	
H16A	0.1098	0.0694	0.4902	0.092*	
H16B	0.1086	0.2465	0.4948	0.092*	
H16C	0.2144	0.1587	0.5050	0.092*	
C17	0.0649 (4)	−0.0666 (7)	0.3986 (2)	0.0462 (15)	
C18	−0.2676 (5)	−1.0395 (7)	0.21641 (19)	0.0508 (16)	
C19	−0.3380 (5)	−0.9630 (7)	0.2361 (2)	0.0519 (16)	
H19A	−0.4084	−0.9654	0.2227	0.062*	
C20	−0.3033 (4)	−0.8837 (6)	0.27553 (19)	0.0435 (14)	
C21	−0.1980 (4)	−0.8850 (6)	0.29602 (18)	0.0405 (14)	
C22	−0.1292 (5)	−0.9617 (7)	0.2757 (2)	0.0549 (17)	
H22A	−0.0588	−0.9621	0.2892	0.066*	
C23	−0.1643 (5)	−1.0374 (7)	0.2354 (2)	0.0570 (17)	
H23A	−0.1177	−1.0871	0.2212	0.068*	
C24	−0.3712 (5)	−1.2088 (8)	0.3953 (2)	0.0524 (16)	
C25	−0.3943 (5)	−1.0715 (8)	0.4093 (2)	0.0643 (19)	
H25A	−0.4533	−1.0587	0.4212	0.077*	
C26	−0.3309 (5)	−0.9481 (7)	0.4061 (2)	0.0578 (17)	
H26A	−0.3466	−0.8535	0.4165	0.069*	
C27	−0.2438 (4)	−0.9654 (7)	0.38723 (19)	0.0430 (14)	
C28	−0.2201 (4)	−1.1102 (7)	0.37496 (19)	0.0500 (16)	
H28A	−0.1604	−1.1255	0.3637	0.060*	
C29	−0.2827 (5)	−1.2322 (7)	0.3790 (2)	0.0554 (17)	
H29A	−0.2654	−1.3290	0.3707	0.067*	
C30	−0.1821 (4)	−0.8338 (6)	0.38025 (18)	0.0380 (13)	
C31	−0.1414 (4)	−0.7112 (6)	0.40758 (17)	0.0373 (13)	
C32	−0.0975 (4)	−0.6191 (6)	0.37874 (18)	0.0386 (13)	
C33	−0.1414 (5)	−0.6905 (7)	0.45721 (18)	0.0589 (18)	
H33A	−0.1783	−0.7728	0.4674	0.088*	
H33B	−0.1748	−0.5964	0.4614	0.088*	
H33C	−0.0712	−0.6891	0.4746	0.088*	
C34	−0.0488 (4)	−0.4708 (7)	0.3863 (2)	0.0466 (15)	

### Atomic displacement parameters (Å^2^)

**Table:**

	*U*^11^	*U*^22^	*U*^33^	*U*^12^	*U*^13^	*U*^23^
Cl1	0.190 (3)	0.0800 (15)	0.0846 (15)	−0.0236 (16)	0.0770 (16)	0.0149 (12)
Cl2	0.0473 (10)	0.0823 (13)	0.0861 (13)	0.0030 (9)	0.0155 (9)	0.0118 (10)
Cl3	0.1092 (16)	0.0820 (14)	0.0773 (13)	−0.0542 (12)	0.0357 (11)	−0.0305 (11)
Cl4	0.1134 (16)	0.0851 (14)	0.0521 (11)	−0.0172 (12)	0.0026 (10)	−0.0250 (10)
Cl5	0.0472 (10)	0.0915 (13)	0.0668 (11)	0.0158 (10)	0.0052 (8)	−0.0112 (10)
Cl6	0.0785 (13)	0.0780 (13)	0.0838 (13)	−0.0437 (11)	0.0093 (10)	0.0142 (11)
O1	0.070 (3)	0.057 (3)	0.044 (2)	−0.018 (2)	0.013 (2)	0.008 (2)
O2	0.076 (3)	0.048 (3)	0.056 (3)	−0.028 (2)	0.007 (2)	−0.002 (2)
O3	0.074 (3)	0.064 (3)	0.059 (3)	−0.028 (2)	0.018 (2)	−0.022 (2)
O4	0.077 (3)	0.055 (3)	0.060 (3)	−0.023 (2)	0.019 (2)	−0.010 (2)
N1	0.043 (3)	0.032 (3)	0.046 (3)	−0.012 (2)	0.012 (2)	0.006 (2)
N2	0.041 (3)	0.046 (3)	0.045 (3)	−0.007 (2)	0.008 (2)	−0.009 (2)
N3	0.036 (3)	0.038 (3)	0.048 (3)	−0.015 (2)	0.004 (2)	−0.005 (2)
N4	0.035 (3)	0.041 (3)	0.039 (3)	−0.009 (2)	0.007 (2)	−0.002 (2)
C1	0.107 (6)	0.043 (4)	0.055 (4)	−0.005 (4)	0.037 (4)	0.003 (3)
C2	0.099 (6)	0.069 (5)	0.042 (4)	−0.004 (5)	0.008 (4)	0.006 (3)
C3	0.072 (5)	0.067 (5)	0.050 (4)	−0.002 (4)	0.016 (4)	0.008 (4)
C4	0.049 (4)	0.036 (3)	0.043 (3)	−0.009 (3)	0.005 (3)	0.002 (3)
C5	0.047 (4)	0.048 (4)	0.061 (4)	−0.006 (3)	0.013 (3)	0.003 (3)
C6	0.072 (5)	0.055 (4)	0.078 (5)	−0.017 (4)	0.044 (4)	−0.013 (4)
C7	0.052 (4)	0.057 (4)	0.047 (4)	−0.027 (3)	0.021 (3)	−0.014 (3)
C8	0.062 (4)	0.035 (3)	0.060 (4)	−0.002 (3)	0.031 (3)	−0.001 (3)
C9	0.037 (3)	0.044 (4)	0.052 (4)	0.001 (3)	0.009 (3)	0.006 (3)
C10	0.036 (3)	0.039 (3)	0.046 (3)	−0.007 (3)	0.015 (3)	−0.002 (3)
C11	0.046 (4)	0.048 (4)	0.061 (4)	−0.013 (3)	0.005 (3)	0.002 (3)
C12	0.045 (4)	0.073 (5)	0.049 (4)	−0.019 (4)	0.006 (3)	−0.004 (4)
C13	0.029 (3)	0.052 (4)	0.039 (3)	−0.004 (3)	0.007 (2)	0.006 (3)
C14	0.034 (3)	0.049 (4)	0.046 (3)	−0.001 (3)	0.010 (3)	0.003 (3)
C15	0.039 (3)	0.044 (3)	0.039 (3)	−0.006 (3)	0.006 (3)	−0.008 (3)
C16	0.078 (5)	0.054 (4)	0.057 (4)	−0.013 (4)	0.027 (3)	0.002 (3)
C17	0.034 (3)	0.049 (4)	0.050 (4)	−0.003 (3)	−0.004 (3)	0.004 (3)
C18	0.070 (5)	0.053 (4)	0.031 (3)	−0.007 (4)	0.013 (3)	−0.007 (3)
C19	0.044 (4)	0.060 (4)	0.045 (4)	−0.007 (3)	−0.006 (3)	−0.001 (3)
C20	0.038 (3)	0.049 (4)	0.044 (3)	0.000 (3)	0.008 (3)	0.012 (3)
C21	0.038 (3)	0.047 (3)	0.036 (3)	−0.010 (3)	0.007 (3)	−0.003 (3)
C22	0.044 (4)	0.077 (5)	0.045 (4)	−0.008 (3)	0.012 (3)	−0.016 (3)
C23	0.057 (5)	0.065 (5)	0.054 (4)	0.000 (4)	0.024 (3)	−0.009 (3)
C24	0.052 (4)	0.059 (4)	0.044 (4)	−0.020 (3)	0.004 (3)	0.020 (3)
C25	0.053 (4)	0.064 (5)	0.083 (5)	−0.016 (4)	0.030 (4)	0.000 (4)
C26	0.053 (4)	0.056 (4)	0.074 (5)	−0.010 (3)	0.033 (4)	−0.005 (3)
C27	0.033 (3)	0.048 (4)	0.044 (3)	−0.005 (3)	0.001 (3)	0.006 (3)
C28	0.038 (4)	0.056 (4)	0.056 (4)	−0.007 (3)	0.009 (3)	0.005 (3)
C29	0.063 (4)	0.046 (4)	0.058 (4)	−0.013 (3)	0.013 (3)	0.003 (3)
C30	0.032 (3)	0.042 (3)	0.040 (3)	0.000 (3)	0.006 (2)	−0.002 (3)
C31	0.037 (3)	0.043 (3)	0.032 (3)	−0.004 (3)	0.007 (2)	−0.007 (3)
C32	0.031 (3)	0.040 (3)	0.044 (3)	−0.010 (3)	0.007 (3)	−0.002 (3)
C33	0.072 (4)	0.064 (4)	0.045 (4)	−0.011 (4)	0.022 (3)	−0.008 (3)
C34	0.038 (4)	0.056 (4)	0.046 (4)	−0.003 (3)	0.010 (3)	−0.001 (3)

### Geometric parameters (Å, °)

**Table:**

Cl1—C1	1.717 (7)	C11—C12	1.378 (8)
Cl2—C5	1.729 (6)	C11—H11A	0.9300
Cl3—C7	1.737 (6)	C12—H12A	0.9300
Cl4—C18	1.735 (6)	C13—C14	1.366 (7)
Cl5—C20	1.725 (6)	C14—C15	1.388 (7)
Cl6—C24	1.739 (6)	C14—C16	1.536 (7)
O1—C17	1.277 (6)	C15—C17	1.453 (8)
O2—C17	1.276 (6)	C16—H16A	0.9600
O2—H2A	0.8500	C16—H16B	0.9600
O3—C34	1.269 (6)	C16—H16C	0.9600
O3—H3B	0.8500	C18—C23	1.360 (8)
O4—C34	1.279 (6)	C18—C19	1.379 (8)
N1—N2	1.336 (6)	C19—C20	1.365 (8)
N1—C13	1.364 (6)	C19—H19A	0.9300
N1—C4	1.426 (6)	C20—C21	1.393 (7)
N2—C15	1.329 (6)	C21—C22	1.376 (7)
N3—C32	1.347 (6)	C22—C23	1.370 (8)
N3—N4	1.349 (6)	C22—H22A	0.9300
N4—C30	1.382 (6)	C23—H23A	0.9300
N4—C21	1.424 (6)	C24—C25	1.337 (9)
C1—C2	1.360 (9)	C24—C29	1.376 (8)
C1—C6	1.379 (9)	C25—C26	1.388 (8)
C2—C3	1.401 (8)	C25—H25A	0.9300
C2—H2B	0.9300	C26—C27	1.394 (7)
C3—C4	1.374 (8)	C26—H26A	0.9300
C3—H3A	0.9300	C27—C28	1.383 (8)
C4—C5	1.397 (8)	C27—C30	1.458 (7)
C5—C6	1.380 (8)	C28—C29	1.377 (8)
C6—H6A	0.9300	C28—H28A	0.9300
C7—C12	1.366 (8)	C29—H29A	0.9300
C7—C8	1.372 (8)	C30—C31	1.393 (7)
C8—C9	1.391 (8)	C31—C32	1.400 (7)
C8—H8A	0.9300	C31—C33	1.501 (7)
C9—C10	1.375 (7)	C32—C34	1.452 (8)
C9—H9A	0.9300	C33—H33A	0.9600
C10—C11	1.384 (7)	C33—H33B	0.9600
C10—C13	1.490 (7)	C33—H33C	0.9600
			
C17—O2—H2A	109.6	H16A—C16—H16C	109.5
C34—O3—H3B	107.3	H16B—C16—H16C	109.5
N2—N1—C13	111.8 (4)	O1—C17—O2	123.6 (5)
N2—N1—C4	117.6 (4)	O1—C17—C15	120.7 (6)
C13—N1—C4	130.5 (5)	O2—C17—C15	115.7 (5)
C15—N2—N1	105.1 (4)	C23—C18—C19	121.3 (6)
C32—N3—N4	103.9 (4)	C23—C18—Cl4	121.7 (5)
N3—N4—C30	112.6 (4)	C19—C18—Cl4	117.0 (5)
N3—N4—C21	117.6 (4)	C20—C19—C18	119.4 (6)
C30—N4—C21	129.0 (5)	C20—C19—H19A	120.3
C2—C1—C6	121.2 (6)	C18—C19—H19A	120.3
C2—C1—Cl1	120.3 (6)	C19—C20—C21	119.7 (5)
C6—C1—Cl1	118.5 (6)	C19—C20—Cl5	120.8 (5)
C1—C2—C3	119.5 (7)	C21—C20—Cl5	119.5 (4)
C1—C2—H2B	120.3	C22—C21—C20	119.9 (5)
C3—C2—H2B	120.3	C22—C21—N4	119.6 (5)
C4—C3—C2	120.3 (7)	C20—C21—N4	120.5 (5)
C4—C3—H3A	119.8	C23—C22—C21	120.0 (6)
C2—C3—H3A	119.8	C23—C22—H22A	120.0
C3—C4—C5	119.1 (5)	C21—C22—H22A	120.0
C3—C4—N1	119.2 (5)	C18—C23—C22	119.7 (6)
C5—C4—N1	121.7 (5)	C18—C23—H23A	120.2
C6—C5—C4	120.4 (6)	C22—C23—H23A	120.2
C6—C5—Cl2	120.0 (5)	C25—C24—C29	121.1 (6)
C4—C5—Cl2	119.5 (5)	C25—C24—Cl6	120.0 (5)
C1—C6—C5	119.4 (6)	C29—C24—Cl6	118.9 (6)
C1—C6—H6A	120.3	C24—C25—C26	120.4 (6)
C5—C6—H6A	120.3	C24—C25—H25A	119.8
C12—C7—C8	120.8 (5)	C26—C25—H25A	119.8
C12—C7—Cl3	120.1 (5)	C25—C26—C27	120.2 (6)
C8—C7—Cl3	119.1 (5)	C25—C26—H26A	119.9
C7—C8—C9	119.1 (6)	C27—C26—H26A	119.9
C7—C8—H8A	120.4	C28—C27—C26	117.6 (6)
C9—C8—H8A	120.4	C28—C27—C30	122.1 (5)
C10—C9—C8	120.7 (5)	C26—C27—C30	120.4 (6)
C10—C9—H9A	119.7	C29—C28—C27	121.6 (6)
C8—C9—H9A	119.7	C29—C28—H28A	119.2
C9—C10—C11	118.9 (5)	C27—C28—H28A	119.2
C9—C10—C13	121.6 (5)	C28—C29—C24	119.0 (6)
C11—C10—C13	119.4 (5)	C28—C29—H29A	120.5
C12—C11—C10	120.5 (6)	C24—C29—H29A	120.5
C12—C11—H11A	119.7	N4—C30—C31	106.3 (5)
C10—C11—H11A	119.7	N4—C30—C27	120.0 (5)
C7—C12—C11	119.8 (6)	C31—C30—C27	133.6 (5)
C7—C12—H12A	120.1	C30—C31—C32	104.2 (4)
C11—C12—H12A	120.1	C30—C31—C33	126.6 (5)
N1—C13—C14	106.4 (5)	C32—C31—C33	129.2 (5)
N1—C13—C10	120.8 (5)	N3—C32—C31	113.1 (5)
C14—C13—C10	132.8 (5)	N3—C32—C34	115.9 (5)
C13—C14—C15	105.3 (5)	C31—C32—C34	130.8 (5)
C13—C14—C16	125.6 (5)	C31—C33—H33A	109.5
C15—C14—C16	129.2 (5)	C31—C33—H33B	109.5
N2—C15—C14	111.4 (5)	H33A—C33—H33B	109.5
N2—C15—C17	118.2 (5)	C31—C33—H33C	109.5
C14—C15—C17	130.3 (5)	H33A—C33—H33C	109.5
C14—C16—H16A	109.5	H33B—C33—H33C	109.5
C14—C16—H16B	109.5	O3—C34—O4	122.7 (6)
H16A—C16—H16B	109.5	O3—C34—C32	119.9 (5)
C14—C16—H16C	109.5	O4—C34—C32	117.4 (5)
			
C13—N1—N2—C15	1.7 (6)	C32—N3—N4—C30	1.1 (6)
C4—N1—N2—C15	177.3 (5)	C32—N3—N4—C21	171.9 (4)
C6—C1—C2—C3	3.5 (10)	C23—C18—C19—C20	−0.1 (9)
Cl1—C1—C2—C3	−176.6 (5)	Cl4—C18—C19—C20	178.7 (5)
C1—C2—C3—C4	−2.4 (10)	C18—C19—C20—C21	−2.0 (9)
C2—C3—C4—C5	−0.8 (9)	C18—C19—C20—Cl5	176.8 (4)
C2—C3—C4—N1	−179.7 (6)	C19—C20—C21—C22	2.2 (9)
N2—N1—C4—C3	67.3 (7)	Cl5—C20—C21—C22	−176.6 (5)
C13—N1—C4—C3	−118.2 (6)	C19—C20—C21—N4	−178.1 (5)
N2—N1—C4—C5	−111.7 (6)	Cl5—C20—C21—N4	3.2 (7)
C13—N1—C4—C5	62.9 (8)	N3—N4—C21—C22	74.7 (7)
C3—C4—C5—C6	2.9 (9)	C30—N4—C21—C22	−116.2 (6)
N1—C4—C5—C6	−178.2 (5)	N3—N4—C21—C20	−105.0 (6)
C3—C4—C5—Cl2	−175.3 (5)	C30—N4—C21—C20	64.1 (8)
N1—C4—C5—Cl2	3.6 (8)	C20—C21—C22—C23	−0.4 (9)
C2—C1—C6—C5	−1.4 (10)	N4—C21—C22—C23	179.9 (5)
Cl1—C1—C6—C5	178.6 (5)	C19—C18—C23—C22	1.9 (10)
C4—C5—C6—C1	−1.8 (10)	Cl4—C18—C23—C22	−176.8 (5)
Cl2—C5—C6—C1	176.3 (5)	C21—C22—C23—C18	−1.7 (10)
C12—C7—C8—C9	−2.5 (9)	C29—C24—C25—C26	2.2 (10)
Cl3—C7—C8—C9	176.9 (4)	Cl6—C24—C25—C26	−177.1 (5)
C7—C8—C9—C10	1.0 (8)	C24—C25—C26—C27	1.6 (10)
C8—C9—C10—C11	0.4 (8)	C25—C26—C27—C28	−4.2 (9)
C8—C9—C10—C13	−178.0 (5)	C25—C26—C27—C30	174.8 (6)
C9—C10—C11—C12	−0.4 (9)	C26—C27—C28—C29	3.2 (9)
C13—C10—C11—C12	178.1 (5)	C30—C27—C28—C29	−175.7 (5)
C8—C7—C12—C11	2.6 (9)	C27—C28—C29—C24	0.4 (9)
Cl3—C7—C12—C11	−176.8 (5)	C25—C24—C29—C28	−3.1 (9)
C10—C11—C12—C7	−1.1 (9)	Cl6—C24—C29—C28	176.1 (4)
N2—N1—C13—C14	−1.8 (6)	N3—N4—C30—C31	−0.5 (6)
C4—N1—C13—C14	−176.6 (5)	C21—N4—C30—C31	−170.0 (5)
N2—N1—C13—C10	−179.9 (4)	N3—N4—C30—C27	175.2 (4)
C4—N1—C13—C10	5.3 (8)	C21—N4—C30—C27	5.7 (8)
C9—C10—C13—N1	40.9 (8)	C28—C27—C30—N4	51.7 (7)
C11—C10—C13—N1	−137.5 (5)	C26—C27—C30—N4	−127.2 (6)
C9—C10—C13—C14	−136.6 (6)	C28—C27—C30—C31	−134.0 (7)
C11—C10—C13—C14	45.0 (9)	C26—C27—C30—C31	47.1 (9)
N1—C13—C14—C15	1.1 (6)	N4—C30—C31—C32	−0.3 (6)
C10—C13—C14—C15	178.8 (6)	C27—C30—C31—C32	−175.1 (6)
N1—C13—C14—C16	−177.9 (5)	N4—C30—C31—C33	−177.6 (5)
C10—C13—C14—C16	−0.1 (10)	C27—C30—C31—C33	7.6 (10)
N1—N2—C15—C14	−1.0 (6)	N4—N3—C32—C31	−1.3 (6)
N1—N2—C15—C17	−177.0 (5)	N4—N3—C32—C34	−176.9 (4)
C13—C14—C15—N2	0.0 (6)	C30—C31—C32—N3	1.0 (6)
C16—C14—C15—N2	178.9 (5)	C33—C31—C32—N3	178.2 (5)
C13—C14—C15—C17	175.3 (5)	C30—C31—C32—C34	175.7 (5)
C16—C14—C15—C17	−5.8 (10)	C33—C31—C32—C34	−7.1 (10)
N2—C15—C17—O1	178.8 (5)	N3—C32—C34—O3	179.8 (5)
C14—C15—C17—O1	3.8 (9)	C31—C32—C34—O3	5.2 (9)
N2—C15—C17—O2	−1.3 (8)	N3—C32—C34—O4	1.3 (7)
C14—C15—C17—O2	−176.3 (6)	C31—C32—C34—O4	−173.3 (6)

### Hydrogen-bond geometry (Å, °)

**Table:**

*D*—H···*A*	*D*—H	H···*A*	*D*···*A*	*D*—H···*A*
O2—H2A···O4	0.85	1.74	2.564 (7)	163
O3—H3B···O1	0.85	1.89	2.723 (6)	165
C16—H16C···Cg6^i^	0.96	3.13	3.867 (4)	135
C33—H33B···Cg4^ii^	0.96	3.29	3.857 (3)	120

Symmetry codes: (i) −*x*+1, −*y*, −*z*; (ii) −*x*+1, −*y*+1, −*z*.
